# The U.S. consumer phosphorus footprint: where do nitrogen and phosphorus diverge?

**DOI:** 10.1088/1748-9326/aba781

**Published:** 2020-10-13

**Authors:** Geneviève S Metson, Graham K MacDonald, Allison M Leach, Jana E Compton, John A Harrison, James N Galloway

**Affiliations:** 1 Department of Physics, Chemistry, and Biology, Linköping University, Linköping, Sweden; 2 National Research Council, National Academies of Science, Washington, DC, United States of America; 3 Pacific Ecological Systems Division, US Environmental Protection Agency, Corvallis, OR, United States of America; 4 School of the Environment, Washington State University, Vancouver, WA, United States of America; 5 Department of Geography, McGill University, Montreal, Canada; 6 The Sustainability Institute, University of New Hampshire, Durham, NH, United States of America; 7 Department of Environmental Sciences, University of Virginia, Charlottesville, VA, United States of America

**Keywords:** sustainability, biogeochemistry, stoichiometry, nutrients, agriculture, food

## Abstract

Phosphorus (P) and nitrogen (N) are essential nutrients for food production but their excess use in agriculture can have major social costs, particularly related to water quality degradation. Nutrient footprint approaches estimate N and P release to the environment through food production and waste management and enable linking these emissions to particular consumption patterns. Following an established method for quantifying a consumer-oriented N footprint for the United States (U.S.), we calculate an analogous P footprint and assess the N:P ratio across different stages of food production and consumption. Circa 2012, the average consumer’s P footprint was 4.4 kg P capita^−1^ yr^−1^ compared to 22.4 kg N capita^−1^ yr^−1^ for the food portion of the N footprint. Animal products have the largest contribution to both footprints, comprising >70% of the average per capita N and P footprints. The N:P ratio of environmental release based on virtual nutrient factors (kilograms N or P per kilogram of food consumed) varies considerably across food groups and stages. The overall N:P ratio of the footprints was lower (5.2 by mass) than for that of U.S. food consumption (8.6), reinforcing our finding that P is managed less efficiently than N in food production systems but more efficiently removed from wastewater. While strategies like reducing meat consumption will effectively reduce both N and P footprints by decreasing overall synthetic fertilizer nutrient demands, consideration of how food production and waste treatment differentially affect N and P releases to the environment can also inform eutrophication management.

## 1. Introduction

Fundamental changes to the global nitrogen (N) and phosphorus (P) cycles, largely driven by increased nutrient use and loss along the food chain, have major social and environmental consequences (Steffen *et al* 2015). Notably, N and P loading are key drivers of surface water eutrophication and associated harmful algal blooms (Carpenter *et al* 1998, Diaz and Rosenberg 2008). These blooms can negatively impact recreation, transportation, fisheries, human health, and water treatment costs (Anderson *et al* 2002). As with the far-reaching social costs of the N ‘cascade’ as reactive N moves through multiple environmental pools (Galloway *et al* 2003, Billen *et al* 2013), excess reactive forms of P associated with human activities can also negatively affect multiple ecosystem services that are vital to human well-being (MacDonald *et al* 2016).

Recent work has highlighted the importance of reducing both N and P inputs to maintain or improve surface water quality (Conley *et al* 2009, Kanter and Brownlie 2019) as well as minimize harmful atmospheric N emissions and associated deposition (Sobota *et al* 2015). Others have highlighted the potential to recover N and P from agricultural and urban waste streams in order to recycle nutrients to agricultural lands, thereby offsetting the need for new N and P fertilizers derived from the energy intensive Haber-Bosch process and non-renewable phosphate rock reserves, respectively (Dumas *et al* 2011, Trimmer and Guest 2018, Powers *et al* 2019). Many actions to reduce N loading likely also reduce P loading, but specific crops, animal products, and management practices have different effects on downstream relative abundance of N and P in the environment (Jobbágy and Sala 2014, Cease *et al* 2015, Bouwman *et al* 2017, Springmann *et al* 2018, Penuelas *et al* 2020).

Both the total amount of nutrients delivered to aquatic systems and the N:P ratio of nutrient loading affects eutrophication (Downing and Mccauley 1992, Martiny *et al* 2014). Given the important role of nutrient stoichiometry (i.e. the ratios of N and P to each other and to other elements) in regulating ecosystem processes and functions (Sterner and Elser 2002), the N:P associated with agricultural inputs, processing losses, and waste warrants further attention. The availability of N and P relative to ecosystem demand could affect both downstream ecosystem response to agriculture as well as the potential to recycle nutrients to meet agricultural fertilizer needs. For example, the Redfield Ratio, a classic stoichiometric ratio in limnology and oceanography based on a constant ratio of carbon, N and P (106:16:1 moles) in marine phytoplankton, can offer insight on nutrient limitation of ecosystem productivity (Klausmeier *et al* 2004). Additional integrated perspectives on how management and consumption patterns in the food system influence the release of N and P to downstream ecosystems is needed.

At least three types of approaches, or lenses, have been applied to estimate the magnitude of nutrient flows in systems, including potential release to the environment. These include: (1) nutrient balances or ‘budgets’, (2) Life Cycle Assessment (LCA), and (3) ‘footprint’ tools. Each of these approaches has strengths and limitations. For example, nutrient budgets aim to quantify major nutrient fluxes in a defined spatiotemporal realm and have been applied to identify major sources and loss pathways of N and P within countries, regions, watersheds, cities, and even a single building (Hong *et al* 2012, Chowdhury *et al* 2014, van Dijk *et al* 2016, Wielemaker *et al* 2019). Yet such studies do not typically evaluate the total impact of a particular product, economic sector, or individual consumer behavior related to nutrient dynamics. In contrast, the purpose of LCA is to quantify the embodied impact associated with nutrient losses to the environment from production or consumption of a product (Finnveden *et al* 2009, Hellweg and Milà i Canals 2014). This provides a more complete view of an activity or sector’s impact on nutrient cycling, but it is of limited use in providing insight into how individuals might alter their behavior so as to mitigate their impact on the environment. Such insight is where footprint tools are particularly useful.

Nutrient footprint tools (e.g. the N-Calculator, Leach *et al* 2012) estimate the amount of nutrients embodied in a product or an entity’s consumption activities. As such, they provide a different perspective than budgeting or LCA approaches. Other percapita N footprint analyses include investigations of N dynamics in multiple countries, institutions, and regions (Gu *et al* 2013, Leach *et al* 2013, Leip *et al* 2014, Stevens *et al* 2014, Galloway *et al* 2014, Shibata *et al* 2014, Hutton *et al* 2017). There have been comparatively few P footprints developed, especially those that mimic the consumer-oriented approach of the N-Calculator. An exception is Li *et al* (2019), who combine country-level fertilizer, trade, and consumption data to quantify per capita P burdens relative to the biophysical limits of the P planetary boundary (which they call ‘P exceedance footprints’). Also, Oita *et al* (2020) explicitly compare N and P footprints focused on release to the environment to gain insight into nutrient cycling, nutrient impacts, and potential nutrient mitigation strategies in three countries in Asia.

Here, we present a new consumer-oriented P footprint tool for the U.S. based on domestic agricultural production systems and dietary patterns, which follows as closely as possible the methods and data sources for N footprints by Leach *et al* (2012), with updated values from Cattell Noll *et al* (2020). We define the P footprint as the amount of P released to the environment associated with the production and consumption of major food groups for one person annually. Our main objective is to estimate how much P is released through the food system for these major food groups in the U.S. from a consumer perspective. We then compare and contrast the N and P footprints for different foods and overall diets. By closely following the N footprint methodology, our results can be used for comparison and assessment of the N:P ratios across different stages of food production and consumption, as well as the N:P of different foods. We address the following questions related to nutrient stoichiometry:

- What is the difference in the magnitude of N and P food footprints? Do different food groups contribute disproportionately to N versus P footprints?
- What are the N:P ratios associated with nutrient release to the environment for different food groups and wastewater?
- How do the N:P ratios of food footprint components compare to known stoichiometric ratios that could influence ecosystem response?

## 2. Methods

The P footprint approach described here emulates the N footprint approach first described by Leach *et al* (2012), with updated methods and values (circa 2013–2015^8^) for N from Cattell Noll *et al* (2020). The N footprint is defined as the N release associated with food and energy consumption, where food consumption is comprised of emissions associated with the food production chain and the treatment of wastewater (including human excreta). Here we focus both N and P footprints only on agricultural production, food processing, food waste, and wastewater components (herein collectively the ‘food footprint’ shown in equation 1)^9^

$$Food\;footprint=release\;via\;wastewater\;+\sum_{food\;items}virtual\;release\times food\;consumed$$

where *releases via wastewater* are expressed as the N or P released per capita per year (see section 2.2), *virtual releases* are expressed as release of N or P to the environment per amount of a nutrient consumed for each food item considered in the diet (see section 2.2), and *food consumed* is the amount of each food item consumed as N or P by one American per year.

Our food N and P footprints center on the average American diet (magnitude and type of different foods consumed) circa the year 2012 as well as the average level of food processing losses, food waste, and wastewater treatment in that period (*sensu* Leach *et al* (2012) and Cattell Noll *et al* (2020)). We consider 17 major crop and animal types in our calculations, with certain crops used to represent broader food categories (table 1, S1, S2, S3 available online at stacks.iop.org/ERL/15/105022/mmedia); for example, the ‘beans’ category is based on soybean data. Potential nutrient release to the environment associated with crop production (e.g. amount of excess fertilizer nutrient applied per crop after accounting for crop harvested removal) is estimated by using recommended fertilizer nutrient application rates and production data from the major producing states for each crop in the U.S. To account for each state’s share of annual production, we calculate a national weighted average for each crop. Recycling of nutrients in the cropping system (e.g. manure nutrients applied to croplands) and in subsequent steps of the food chain (e.g. byproducts of crop processing) is accounted for by iteratively running a model of releases that calculates the recycled ratio of nutrients from each stage that enters as an input to the next stage (see Cattell Noll *et al* (2020) for details, and table S1 for an equation representation of P that follows how the N food footprint method is presented). Because our focus is on terrestrial agriculture, we do not include a P footprint for fish consumption although there are P losses associated with aquaculture that draw from terrestrial feed sources, such as oilseed crops (Fry *et al* 2016, Huang *et al* 2020). Adding seafood is a next step in developing the P footprint, noting that there are many methodological details that must be considered (Oita *et al* 2016). However, we note that seafood (including fish) accounted for less than 5% of both per capita protein and calorie supply in the U.S. in 2012 (FAOSTAT 2020) and only 3.4% of the total consumption + production food N footprint (Cattell Noll *et al* 2020).

**Figure 1.**
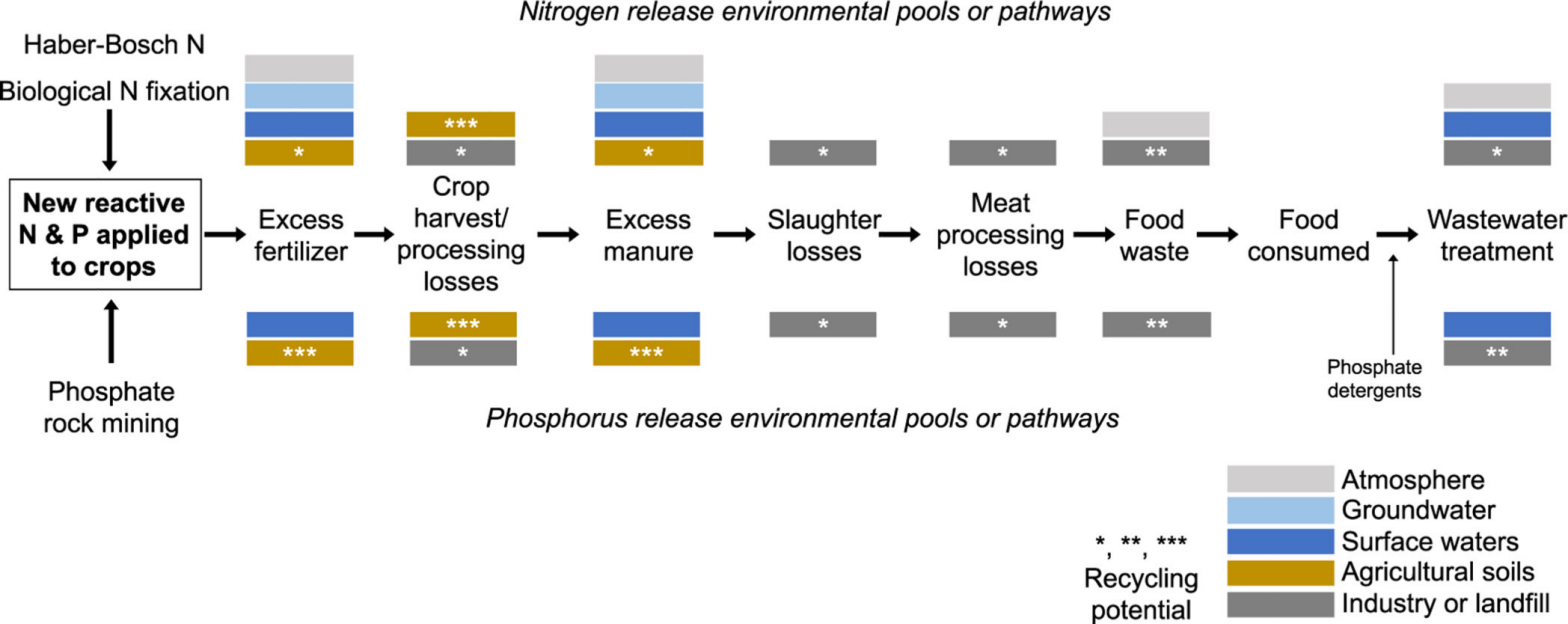
Major differences in the input and release pathways for N and P along the food production and consumption chain. Boxes and arrows represent the major steps accounted for in the N and P footprint methodology used in this paper. The top represents N, and the bottom P, releases to the main receiving system at each step, where colors indicate the system type. Grey is atmosphere, light blue is groundwater, dark blue is surface water, brown is agricultural soils, and dark grey is industrial and landfill systems. Asterisks (∗, ∗∗, ∗∗∗) indicate our qualitative assessment of recycling potential for N or P flows to be returned to the food production system (in the same or a subsequent year) associated with these major flows. Smaller potential release/recycling pathways not included here include P release to groundwater and nutrient recovery from water discharged from wastewater treatment plants.

While the methods for calculating the P footprint is similar to those previously used to calculate the N footprint, there are some important conceptual (figure 1) and methodological differences (section 2.2 and tables 1, S1, and S2). For P, the potential for internal recycling within agricultural systems is considerably higher than for N (Sattari *et al* 2012, Rowe *et al* 2016). As a result, P fate and impacts differ considerably from those of N. Notably, whereas N can be transformed and lost from soils as a gas via denitrification (as well as ammonia volatilization and other reactive forms), there is no significant gaseous loss pathway for P.

### 2.1. N footprint approach

Details on the N footprint approach are provided in Leach *et al* (2012) and updated in Cattell Noll *et al* (2020). Coupling between food intake and N release is done through a virtual N factor (VNF), which estimates the amount of N release along the food chain (*i*) per unit of N consumed in each food item and then can be expressed (*ii*) per unit of food consumed (kg basis). For crop VNFs, total N release is estimated based on the application of fertilizer (and biological N fixation), crop N uptake and harvest removal, processing, and consumption, while also accounting for recycling within and across each stage. For animals, VNFs require information about both losses directly associated with manure excretion over the animals’ different growth stages, slaughter, and processing, and also integrated feed crop VNFs to estimate losses associated with crops grown to feed animals over different life stages.

### 2.2. P footprint approach

**Figure 2.**
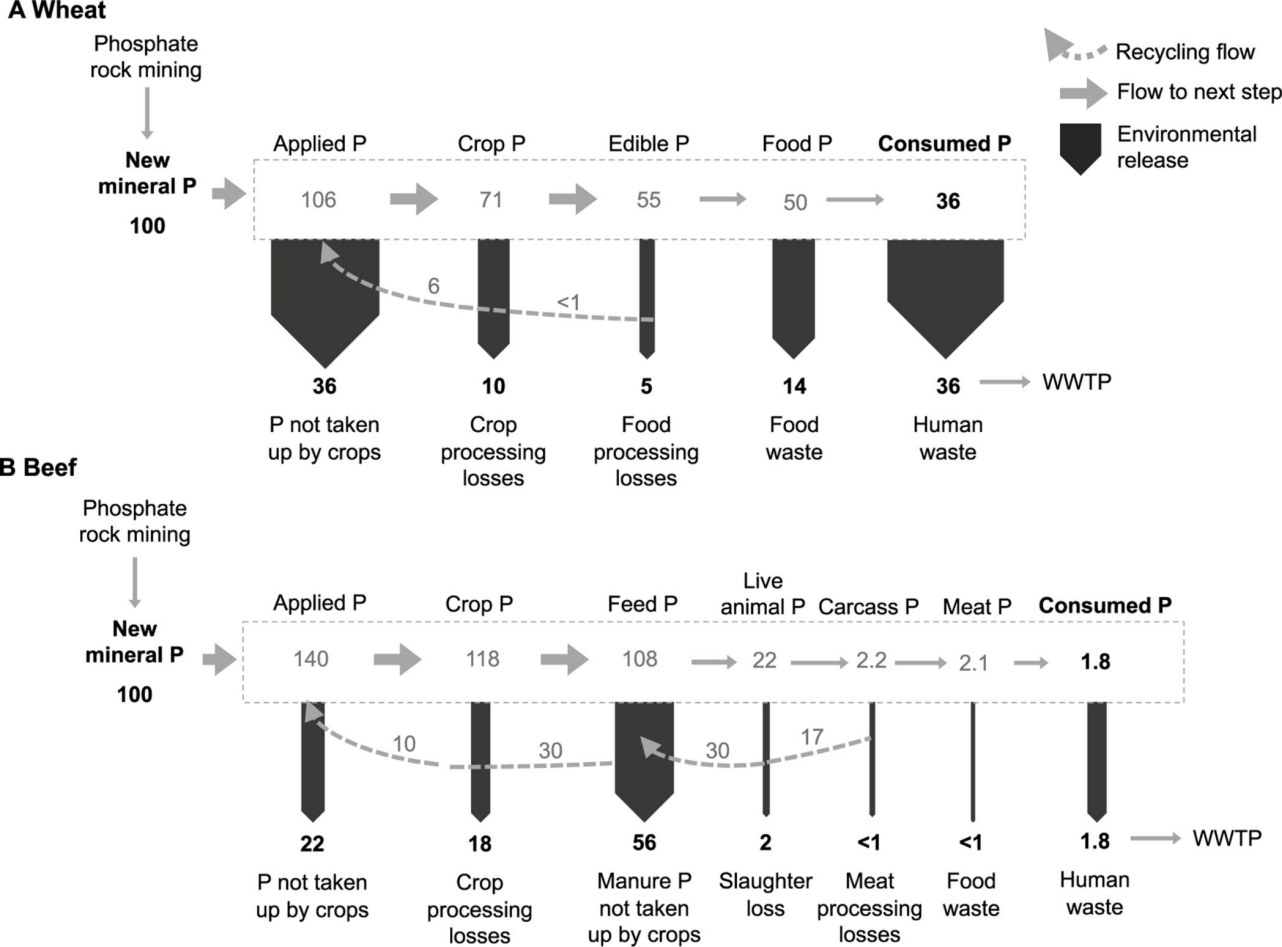
Schema representing major P flows from the food system accounted for by the virtual phosphorus factors (VPF) for wheat and beef. Here, we track the fate of 100 units of ‘new’ mineral P applied to domestic croplands in the U.S. for a given product. Panel (A) shows wheat as an example of the largest VPF for crop products and panel (B) shows beef as an example for the largest VPF in animal products. Grey solid arrows are proportional to the amount of P moving to the next step, dotted grey arrows represent recycling back into the food system, while black solid downward arrows indicate the amount released from the system. Each VPF is calculated by dividing the total amount of P released before consumption (sum of black arrows before human waste) by the amount of P consumed (for wheat, the VPF is 1.8 = 64.6/35.5; for beef, the VPF is 54.9 = 97.9/1.8). The numbers from the figure may not match the VPF exactly due to rounding. Accounting for recycled flows cause applied P to be greater than the new mineral P added; the model which produces the numbers depicted here is run for multiple iterations to account for potentially asynchronous redistribution across steps through use of recycled P (e.g. byproducts and waste).

We follow the same key steps to calculate virtual P factors (VPF) as for VNFs (figure 2, equation (2), table S1), in addition to using the same data sources whenever possible (tables 1 and S2). As with N, we calculate VPFs standardized (*i*) per unit of P consumed in each food item and then (*ii*) per unit of food consumed (kg basis), which can then be multiplied by the amount of each food consumed per individual to calculate the total P footprint for an average U.S. consumer. However, in addition to different environmental release pathways, an important distinction between N and P relates to how they are allocated in biological tissues. Since protein is 16% N, knowing the protein content of crops, animal feeds, and human diets facilitates the calculation of N release once waste and loss factors are known. There is no comparable shortcut for P. The following sections explain where changes needed to be made to calculate the VPFs:

$$Virtual\;release\;of\;P\left(VPF\right)=\frac{P\;release\;at\;field+crop\;waste+processing\;waste+food\;waste\;+manure\;release+slaughter\;release}{P\;consumed\;in\;food\;item}.$$

Note that releases for manure and slaughter are only applicable to animal products, and these releases specifically refer to the ‘loss’ not recycled to meet crop needs as per Cattell Noll *et al* (2020). Also, for animal products *P release at field* refers to releases associated with feed production.

#### 2.2.1. Food consumption

Because P intake cannot be estimated from protein consumption, we use a national database on U.S. food consumption patterns (by weight and calories) that accounts for food loss (USDA ERS 2019) instead of national average protein consumption reported in the FAOSTAT database [used by Leach *et al* (2012) to estimate the N footprint]. We converted from total food weights to P amounts on a per-crop or per-food item basis, following USDA (2017).

#### 2.2.2. Crop production

For crops, we closely follow the VNF method to calculate crop-specific VPFs. This involved collecting fertilizer P application rate recommendations for particular crops following similar sources (e.g. university extension agencies) as Cattell Noll *et al* (2020) for N (see tables 1, S1 and S2); we then weighted these P rates by state-specific crop production totals to calculate a national average. By comparing P application to the amount of P that ends up in the harvested crops and crop residues, we estimate excess P potentially released to the environment (as per steps in figure 2). We acknowledge that actual fertilizer use may deviate from state-level recommendations; as a simple test of this, we examined the case of corn, which is by far the largest crop in the U.S. in terms of area, production, and P fertilizer use (Metson *et al* 2016b). We compared the national P fertilizer application estimated in our model (*Σstate-level P fertilizer rate recommendation* × *state-level corn area planted*) for 29 states to the actual fertilizer use for corn nationally in the U.S. in 2014 (USDA ERS 2019) and found very close agreement for this crop (i.e. ∼904 000 tonnes P yr^−1^ compared to ∼906 000 tonnes P yr^−1^, respectively). Data on crop-specific fertilizer application rates however are only available for a few select crops which is why we use recommended application rates. We caution that our estimates for more minor crops or crops in different years may deviate further.

#### 2.2.3. Animal production

A key step in estimating the footprint of animal products is to estimate feed inputs required over an animal’s key growth stages. To estimate these, we draw from national recommendations for animal feed rations (table 1). As with human food consumption, animal P intake requires some special considerations. Since the P ingested by some monogastric animals is not completely digestible and some animals receive mineral P supplements, the recommended daily intake (RDI) of feed may not accurately reflect annual P ingestion. Sometimes animal P requirements are met by adding mineral supplements to the feed, but here we assume that P requirements are fulfilled by crops only. MacDonald *et al* (2012) estimate that less than 5% of annual mineral P use in the U.S. goes to feed supplements, comprising up to 12% of total P intake by livestock animals, particularly for poultry. Feed supplement use is mainly due to the un-digestibility of P in the form of phytate by chickens (Ceylan *et al* 2003). To account for this discrepancy between the amount of feed intake and P recommendations, we therefore converted recommended broiler and layer chicken feed ratios to a total P intake basis (table 1). Although phytate availability can also be a problem for pigs, recommended feed intake was directly reported as total P already (NRC 2012).

#### 2.2.4. Wastewater^10^

We assume that all P consumed by humans as food is ultimately released as excreta to the sanitation system, since adults typically retain <1% of P intake in their bodies (Cordell *et al* 2009). The per capita amount of P released to the environment after factoring in wastewater treatment was modified from Metson *et al* (2017) to better match Cattell Noll *et al* (2020). We account for average P excreted and industrial detergent use per capita, U.S. national average sewage connection rates, and the coverage of primary, secondary, and tertiary treatment plants across the country in 2012 and their respective P removal efficiencies. More specifically, the U.S. EPA (2012) Community Watershed Needs Survey indicates the percentage of the U.S. population connected to centralized sewer systems that are covered by either no discharge (7%), advanced (54%), secondary (38%), and less than secondary treatment (2%) facilities. Since estimates of households relying on decentralized options (e.g. septic, cesspools, chemical toilets, simple pit latrines) are uncertain, we assume that 76% of the U.S. population are connected to central systems based on EPA (2012) and the remainder are from decentralized systems. These coverage fractions were then paired with the P removal efficiencies associated with each broad category of centralized wastewater treatment (10% for primary, 30% for secondary, 90% for advanced and 100% for no discharge, Morse *et al* (1993)) and septic as a representative of for those relying on decentralized systems (50% (Schellenger and Hellweger 2019)). While only 25% of P settles at the bottom of septic tanks (Lusk *et al* 2017), further retention happens in the drainage field where effluent is treated; retention for on-site septic and cesspool systems has been estimated between 50% and 75%, where, overtime, soil P can leach towards waterways (Schellenger and Hellweger 2019). Overall, we assume that 63% of P produced per capita is retained based on the combination of data described above. The values and approach used here are similar to other U.S. (Hale *et al* 2015) and global (Van Drecht *et al* 2009) studies looking at both N and P in wastewater. Still it is important to acknowledge that specific retention values and treatment technologies vary widely across the U.S., depending a on diverse factors including infrastructure types and prevalent industries, which in turn impact nutrient concentrations in waterways (EPA 2012, Skinner and Maupin 2019); decentralized system retention values likely vary even more as management practices and biophysical conditions like soil type, groundwater connectivity, and seasonality can affect P release to the environment (Lusk *et al* 2017).

### 2.3. Sensitivity analysis

We conduct a basic uncertainty analysis for key input parameters to provide insight on the range of variability. For N, we use the 95% confidence intervals developed for ‘conventional agriculture’ as shown in Cattell Noll *et al* (2020). For P we follow the same methods and data sources, the only exception being a slight deviation in the number of states used for fertilizer recommendation rates available for P (as presented in table S2). For crops, the 95% confidence interval related to the variance in the ratio of P fertilizer applied to P in plant uptake and harvested yield. In other words, the upper and lower values give a range of VPF values that account for the variation in fertilizer recommendations across states as well as the larger uncertainty associated those crops where few states (and/or a small amount of acreage) had available data. For animal products, this uncertainty range is quantified by combining the variability in fertilizer recommendations for feed crops as well as that of the feed conversion ratio (FCR). This is the amount of feed required to produce one kg of meat, milk, or egg. 95% confidence intervals were calculated using a restricted maximum likelihood estimation with the *lme4* package in R 3.6.2 (R Core Team 2019). No new data is introduced to create ranges, as these calculations only account for state variability in fertilizer application rates and yields, as well as reported values for feed conversion efficiencies.

### 2.4. N:P ratio of virtual nutrient factors and footprints

In order to compare the release of N to the release of P associated with different footprint components we use three metrics: (*i*) the total g of nutrient release per kg N or P consumed, (*ii*) the N:P ratio of release per kg N or P consumed, and finally (*iii*) the N:P ratio of release associated with a 100-unit addition of’new’ nutrient to the system. The first metric gives a sense of both the absolute and relative release values associated with the full food production chain for each food group. The ratios, on the other hand, rescale the issue and allow us to look at potential health, environmental, and recycling implications of the footprints. The N:P ratios are calculated by dividing N release by P release to express the kg of N released for every kg of P released (i.e. not on a molar basis). We compare the N:P ratios per kg of specific food types to one another and to literature values. For instance, we use the Redfield ratio as a benchmark for water quality implications. We also pay special attention to comparisons between human consumption and RDI (Cease *et al* 2015), and between sewage release and other types of treatment such as septic (Downing and Mccauley 1992). The final metric, (*iii*) N:P release per 100 units of new nutrient input, follows release for beef and wheat production (as in figure 2) and allows us to consider how the release associated with production steps for a particular food type might give us the information required to maximize recycling and minimize losses to waterways (see figure 1).

## 3. Results and discussion

**Figure 3.**
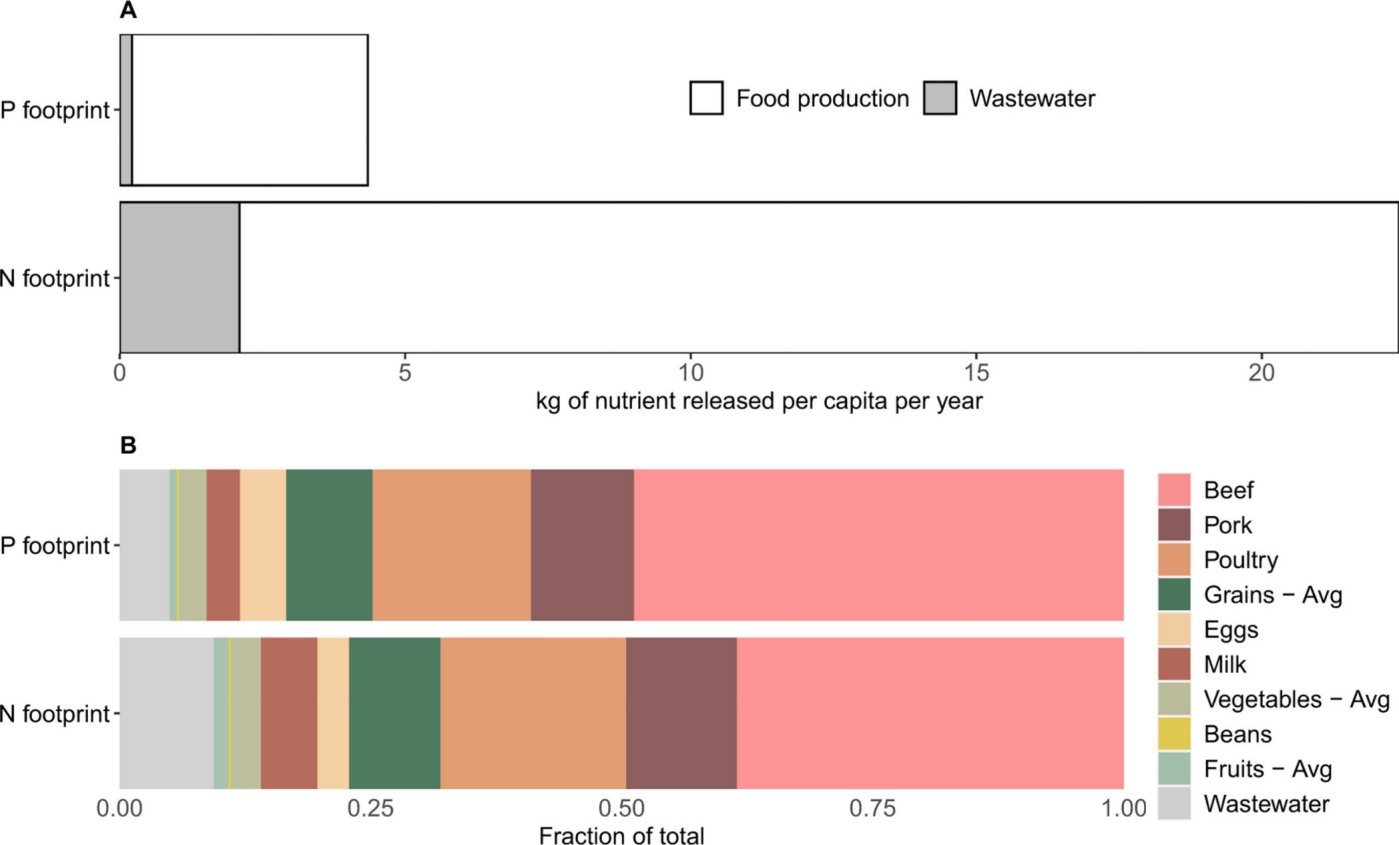
Total P and N footprints associated with the annual consumption of foods in the U.S., including per capita wastewater discharge for an average American resident.

We estimate the average U.S. consumer’s total annual P footprint to be ∼4.4 kg of P capita^−1^ yr^−1^ released to the environment, which is smaller than the food component of the total N footprint of ∼22.4 kg N capita^−1^ yr^−1^ (Cattell Noll *et al* 2020). This disparity between the absolute N and P footprint magnitudes (figure 3(A)) is expected considering that relatively smaller amounts of P inputs are typically required in agricultural system compared to N because of plant stoichiometric needs as well as management practices. In fact, anthropogenic N mobilization has been much higher than P in an absolute and relative terms globally (e.g. due to biological N fixation and atmospheric deposition pathways for N, as well as fertilizer use (Penuelas *et al* 2020)). The proportion of the N and P footprints attributable to different foods are largely consistent for N and P but meat contributes a greater fraction to the P footprint than to the N footprint. Animal products comprise 78% of the average per capita P footprint and 72% for N. Beef is the single largest contributor to both N and P footprints, although beef does contribute proportionally more to the P footprint. This trend is in line with multiple previous studies that have shown the disproportionate environmental impacts of beef (Poore and Nemecek 2018, Clark *et al* 2019), largely due to the relatively poor feed conversion efficiency compared to other animal products and relative inefficiency from a food systems perspective compared to directly consuming crops in human diets (Cassidy *et al* 2013). After beef, the largest difference in proportional contributions to the footprints comes from wastewater. 5% of the annual per capita P footprint can be attributed to surface water release following wastewater treatment (which includes human excreta as well as industrial detergents); in comparison, N removal via wastewater treatment is less efficient than wastewater P removal, contributing 9% of the N footprint.

### 3.1. VPFs and VNFs per unit of food consumed

**Figure 4.**
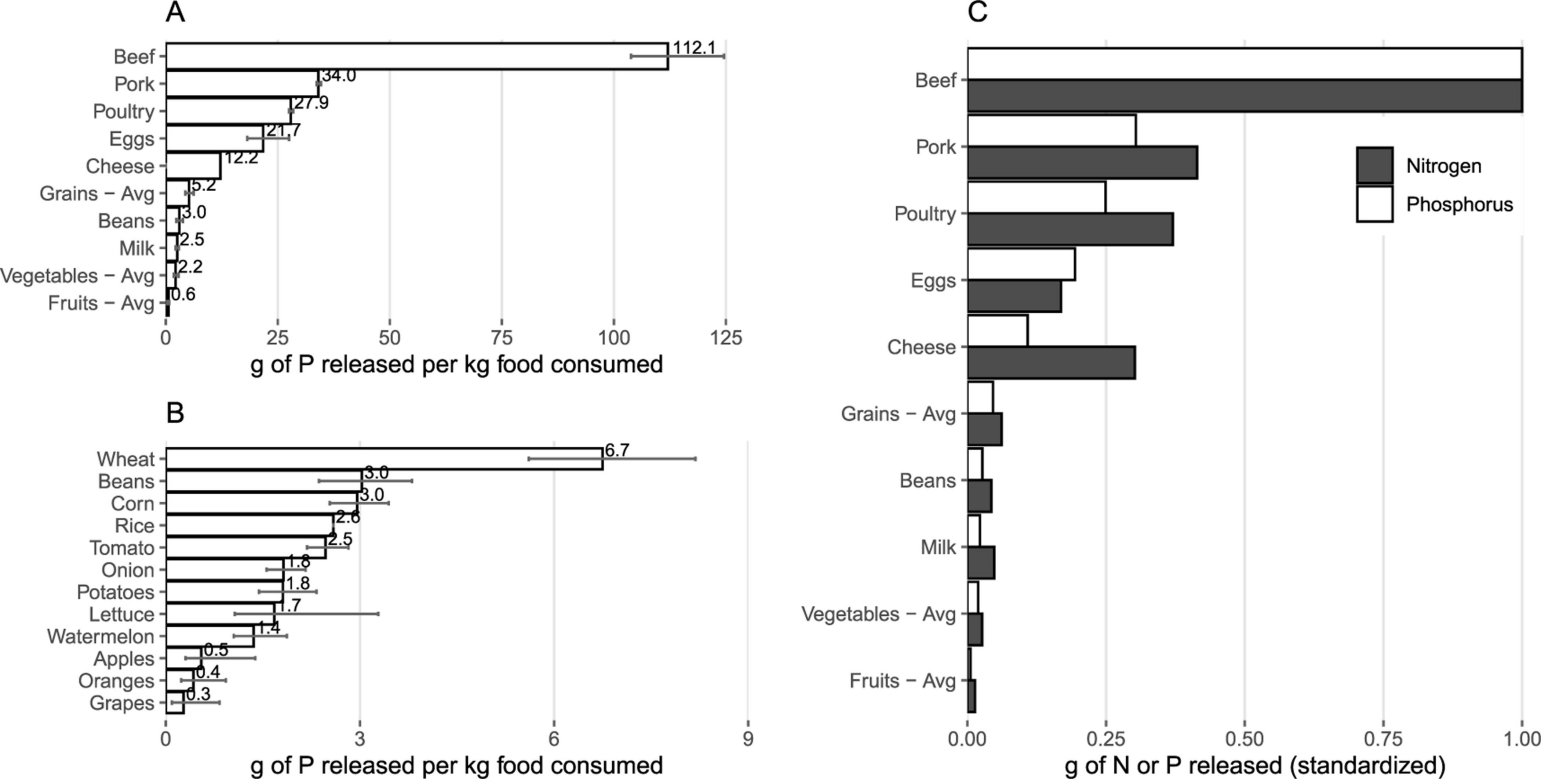
P released to the environment for (A) animal products and vegetal product food groups, (B) specific food items that comprise the vegetal product food groups presented in A, and (C) in comparison to N released to the environment standardized by dividing each food by the value for beef, where P is represented as white bars, and N is represented as dark grey bars. Error bars in (A) and (B) represent the 95% confidence intervals related to the variance in crop fertilizer recommendations and animal feed conversion ratios. Note that the results in figure 3 are obtained by multiplying the values presented here by the amount of each food group consumed by a person in 1 year. P released values used here are also presented in table S4.

Overall patterns of nutrient release to the environment per kilogram of food consumed are largely consistent for N and P, with lower nutrient releases for crops than animal products and large nutrient footprints for beef (figures 4 and S1). One kg of beef consumption is associated with ∼112 g P that does not end up in consumed foods. This impact is more than three-fold that of the next largest food type^11^ (pork, with ∼34 g P released per kg of food consumed). Beef also has a much larger N footprint than other animal products. Although cheese is both P and N intensive (ranking as the fifth and fourth largest for P and N, respectively), milk has a lower P footprint relative to grains (ranking as the eleventh largest). Fruits have the lowest N and P footprints compared to other vegetal foods (fruits < vegetables < beans < grains, for both N and P). Beans have a mean N and P footprint slightly greater than vegetables, but smaller than that of most grains. Animal products generally have larger P footprints than plant products, in part because they have more opportunities for leakage along production chains. This is illustrated by the beef versus wheat comparison (figure 2). Milk is an exception because milk has a relatively low P content and cows produce a lot of milk over their lifetime (which means the P release per kg of product is low). When multiplied out to reflect annual milk consumption (figure 3) in diets it becomes a more substantial part of the total U.S. footprint.

Our sensitivity analysis suggests that these overall trends across food products are robust. Confidence intervals around the P release values do not overlap among animal products or among grains, beans, and vegetable and fruits as category averages (figure 4(A)). This suggests that these relative rankings would likely not change due to state-by-state variability in fertilizer N/P application recommendations or changing the FCRs used in calculating VPFs of animal products (figure 4). The exception is milk, which overlaps with the confidence interval for vegetables.

### 3.2. VPFs and VNFs per unit of nutrient consumed

Our base calculation for the VNFs and VPFs is on a nutrient consumption basis, which helps account for potential effects of variation in protein and P content of foods (i.e. kg N or P released per kg N or P consumed in foods). When we compare the nutrient released per kg of nutrient consumed, the order of food groups changes, with more distinct patterns between the two nutrients (table S3 and figure S2). For example, while the VPFs of pork and poultry per unit P consumed rank second and third (note the overlapping confidence intervals), they rank as fifth and eighth largest, respectively, for the VNF. Nonetheless, beef still has the largest VNF and VPF with this alternate standardization.

### 3.3. Comparison of P footprint results with other studies

#### 3.3.1. Comparison to studies with U.S. results

Our P footprint results for the U.S. have a similar order of magnitude and relative ranking across foods as other approaches. Metson *et al* (2012) found that the amount of mineral P fertilizer needed to produce food for one U.S. consumer is ∼6.9 kgP capita^−1^ yr^−1^, with 83% of this footprint linked to animal products. This fertilizer use footprint is higher than the release P footprint reported in this study (∼4.3 kg P capita^−1^ yr^−1^, figure 3) but with a very similar share from animal products (78%). The Metson *et al* (2012) definition was based on 2007 globally weighted production systems but was conservative in that it assumed that grazing land did not receive mineral fertilizers. Here, we assume that all food consumption was sourced from U.S. domestic production, which could partially explain the lower value, since fertilizer and food imports have an important role in the total P released to the environment globally (MacDonald *et al* 2012, Lun *et al* 2018, Sun *et al* 2019). The U.S. has one of the highest P planetary boundary (threshold as defined by Steffen *et al* (2015)) ‘exceedance’ footprints as a nation (724 kt P), and is relatively high per capita (2.33 kg P); although it is a major export-oriented agricultural nation, its contribution from imported food is roughly equivalent to the exceedance associated with production in the U.S. for export (Li *et al* 2019).

#### 3.3.2. Comparison to other P footprints in other countries

The overall P footprint values reported here are of comparable magnitudes to those of India, China, and Japan (1.6–6.1 kg P capita^−1^ yr^−1^ in 2013) using a similar approach (Oita *et al* 2020). However, vegetables and cereals comprise a much larger share of the footprint in these countries than in our U.S. footprints. Lower per capita meat consumption in these countries compared to the U.S. likely accounts for some of this discrepancy (West *et al* 2014). Differences in agricultural production practices and data likely also play an important role. For instance, the meat product VPF (kg P released per kg P consumed) ranged from 6.05 in China and up to 31.03 in India (Oita *et al* 2020), and the average U.S. VPF for meat products in our study is 28.4. Such differences may be arising from different feed crop fertilizer practices, feed composition, animal breeds, or differences in data specificity (e.g. we used U.S. specific values while Oita *et al* (2020) used FAOSTAT data to allow for inter-country comparisons). As with meat, our milk VPF value is within the ranges found in these large Asian countries (Oita *et al* 2020). However, our VPF for milk production in the U.S. is surprisingly low (∼2.5 kg P released per kg P consumed in milk) considering relatively intensive dairy production practices in the U.S.; we attribute this to the low P concentration of milk and the high productivity of animals. Finally, while our P and N footprints are based on average national data on production and consumption patterns for the circa 2012–2015 period, these values can vary considerably sub-nationally and over time.

### 3.4. N:P ratio of the food footprint and its components

**Figure 5.**
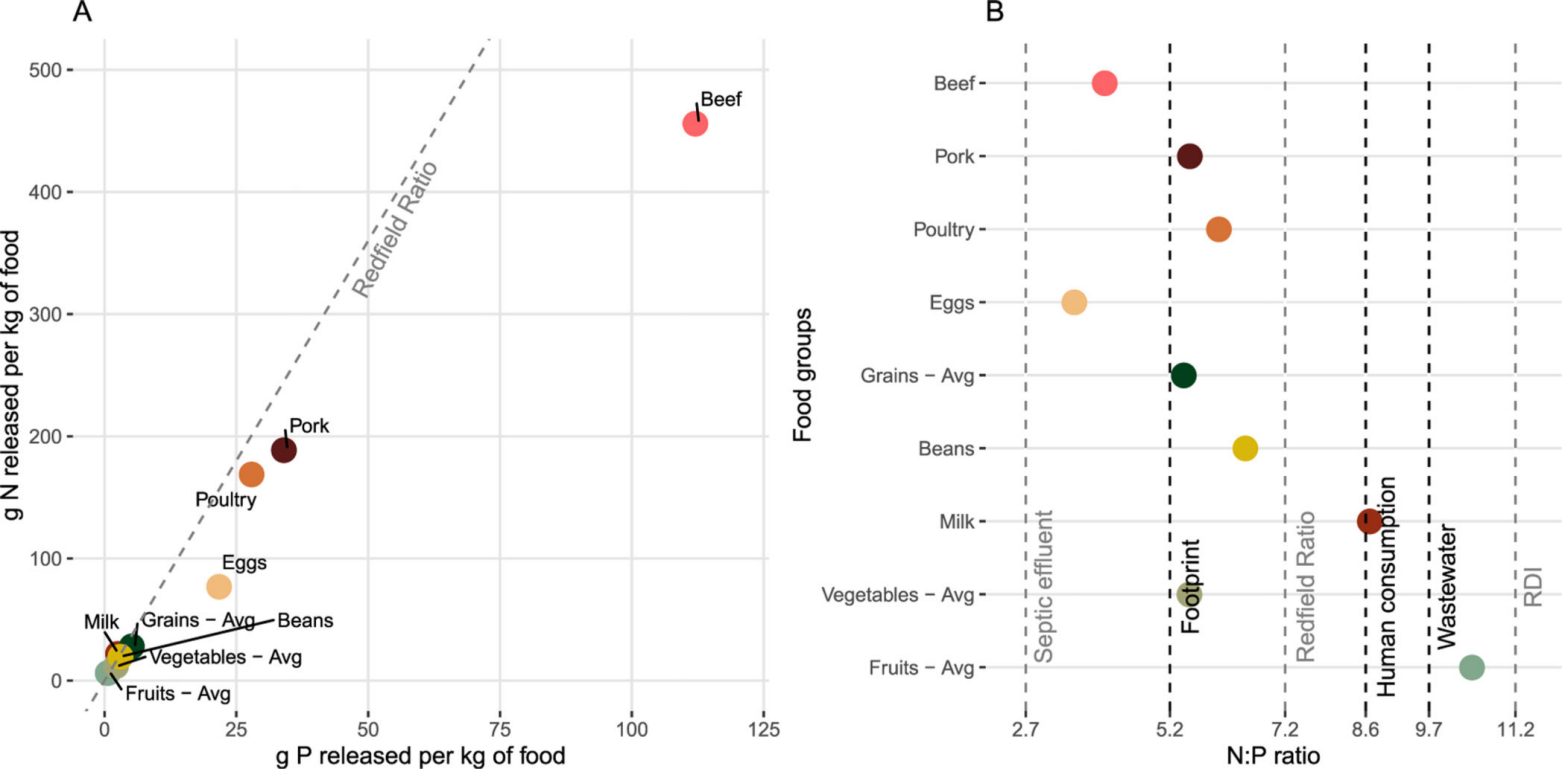
Relationship between N and P release associated with U.S. VNFs and VPFs among major food groups. The VPFs and VNFs are expressed as (A) grams of P released vs grams of N released per unit (kg) of each major food group, and (B) as an N:P ratio (i.e. a ratio of nutrient release to the environment per kg of food). In panel B food groups are ordered top to bottom according to their P release value to match the order in panel A and figure 4. Reference values are expressed as dotted lines where black lines are values calculated and presented in this study and grey lines indicate values from the literature. More specifically these include septic effluent (Downing and Mccauley 1992), the per capita footprints (as per figure 4), the Redfield ratio (Downing and Mccauley 1992), the amount of nutrients consumed calculated (see methods), wastewater (figure 3), and finally the recommended daily intake of nutrients (RDI, Cease *et al* 2015).

For every kg of P released to the environment for food, the average U.S. consumer releases ∼5.2 kg of N, but there is a large amount of variability in the ratio among food groups, and even more so between food consumption and wastewater (figure 5). Because beef and other meats result in so much more N and P release per unit food consumed, at first glance it can seem like other foods are similar to one another and close to the Redfield Ratio (figure 5(A)); however our results indicate that grains and pork have similar N:P release ratios (figure 5(B)). While fruits and milk have N:P ratios of 10.4 and 8.8, respectively, all other foods are between 3.5 (eggs) and 6.5 (beans), meaning that they release relatively more P than N compared to relevant ecological and heath values. In other words, the cumulative release associated with these food items are lower than what phytoplankton require to grow *(Redfield ratio)*, what the average human does consume *(human consumption)*, and what they should consume *(RDI)* in the U.S. (see figure 5(B)). The human consumption N:P ratio maybe below the RDI because even though American’s consume more protein (and thus N) than is needed, they proportionally eat more P than is recommended (Cease *et al* 2015).

Interestingly, the ratios associated with human waste are highly variable (seen as difference between wastewater and septic dotted lines in figure 5(B)) and do not closely match that of any food group or recommended dietary consumption. We estimate that wastewater release to the environment has a higher N:P ratio than septic waste, given the mix of centralized systems and septic systems across the U.S.. It could be that N entering waste treatment plants is nitrified and then released to surface waters and requires tertiary treatment to remove nitrate (Holmes *et al* 2019). In contrast, the septic environment is designed to maximize denitrification, and thus may have a lower N:P ratio than the sewage (Wilhelm *et al* 1994); although both N and P losses from septic towards waterways can be high (Iverson *et al* 2018).

**Figure 6.**
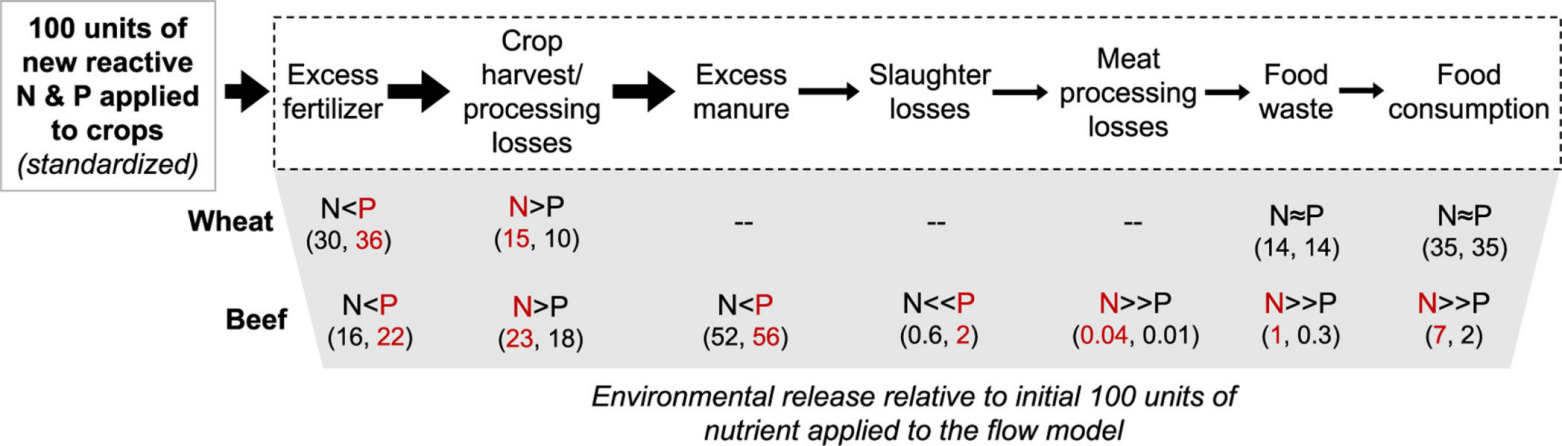
Relative release of N and P across stages of the food supply chain from our VPF models compared to those for the VNF models by Cattell Noll *et al* (2020), using wheat and beef as examples of key crop and animal products, respectively. The numeric values are based on tracking 100 hypothetical units of new mineral N or P applied to the model and compare the relative release to the environment at each step after calculating flows to the next step and recycling. The steps depicted, and the values for P, match those presented in figure 2. The larger relative amount of nutrient released (either N or P) is indicated in red as well as by greater than (>) or less than (<) and approximately equal (≈) signs. We use much greater than (≫) signs when relative differences are particularly pronounced for N and P even though absolute differences may be small given our focus on potential stoichiometric implications in downstream environments.

As is the case with different food groups overall (figure 5), the relative release of N and P at each step of the production chain varies for individual foods (figure 6). For every 100 units of new N and P there is relatively more release of P from excess fertilization (P > N for fertilizer and manure recycling), postharvest crop losses have relatively more N than P release for both wheat and beef. The larger P releases related to manure and slaughter processing in beef are expected because most P is in manure and bones instead of in the meat, whereas more N is found in meat because of its protein content. This also explains why losses of N due to food waste and consumption of beef are relatively greater for N than for P in comparison to wheat (i.e. food waste and consumption have similar impacts on N and P footprints). Although many release pathways can be opportunities for recycling, animal manure and carcasses are particularly important potential P recycling pathways (figure 1).

In summary, the average U.S. consumer’s diet is less efficient in its use of P than N along the full production-to-waste chain (prior to wastewater treatment), and this is in large part because of the releases associated with animal products (except milk, figure 5). However, the decoupling of N from P due to sewage and septic treatment means that the average U.S. consumer releases relatively more N than P to waterways relative to what they consume (figures 5 and 6). If the U.S. replaces septic with connections to central sewer systems it is possible that the discrepancy between N and P removal becomes even more accentuated. For instance more effective removal of P than N from wastewater treatment plants over time has been observed in China, where urban lakes have experienced increasing TN:TP ratios as cities ‘improved’ to municipal wastewater treatment (Tong *et al* 2020). The specific values of nutrient ratios (wastewater or other), and the ecological implications of any of these stoichiometric relations likely varies widely across the country. Similarly, the relative release of N and P at different steps along the production chain (figure 6) for different food items will both affect recycling potential and have ecological impacts.

### 3.5. Implications for management

Changes in production practices, diet, and waste management could change not only total amounts of N and P released, but also their ratios; both of which affect ecosystems. Our results indicate that the N:P ratio released in relation to different food items (figure 5), and the relative release of N and P at different production steps (figure 6), varies by an order of magnitude. Following are three examples of how stoichiometry should be taken into consideration for management.

#### 3.5.1. Production practices

When recycling manure back to agricultural lands, crop responses are not only responsive to total nutrient addition, but importantly N:P ratios (Sileshi *et al* 2017). Relatively more P is released as manure (figure 6) and this makes it a highly recyclable release pathway, while N may be lost to the atmosphere (figure 1). Often N becomes the limiting nutrient, and as such P recycling can contribute to soil storage and release to the environment instead of food security. In fact, both new mineral and recycled P that is applied to agricultural fields but not taken up by crops in harvest removal is prone to long-term buildup in soils and sediments (Sharpley *et al* 2013, Haygarth *et al* 2014). P released to surface water is often a small fraction of annual inputs, but when P is mobilized, it can be recycled from sediments creating positive feedbacks that lock-in eutrophication and algal bloom problems even if inputs are reduced (Lürling *et al* 2016, Carstensen and Conley 2019). On the other hand, water bodies where terrestrial nutrient management have been very effective for P can switch to N limitation (Bouwman *et al* 2017). In other words, both N and P should be managed together, to reduce the likelihood that either is in excess and lost to the environment

#### 3.5.2 Diets

Consumers eating fewer animal products would decrease the total release of both N and P to the environment (figures 3–5). In addition, moving towards more plant-based diets would decrease demand for mineral P fertilizer; however such diets also increase losses to waterways from human excreta as plant based protein sources often contain more P than animal ones (Metson *et al* 2016a, Forber *et al* 2020). This means that careful planning needs to take place to ensure waste management infrastructure can keep up and avoid aquatic pollution. Current wastewater infrastructure in the U.S. removes relatively more P than N (figure 5), but total treatment capacity is also important to consider as upstream behaviors change.

#### 3.5.3. Waste

Sewage, depending on treatment type, had the widest variability of N to P release and was not systematically linked to the ratio of consumed N and P in food (figure 5, Cease *et al* 2015); ergo, waste treatment technology choices can have a large impact on N:P ratios of sewage from human settlements and, as a result, water quality. A wide diversity of technologies are available to increase the recovery (and reuse) of nutrients, especially P, from human excreta and wastewater more generally (Harder *et al* 2019), and increasing access to advanced sanitation across the U.S. is necessary (EPA 2012, Skinner and Maupin 2019). Implementation of waste management solutions should consider both total nutrient recovery capacity and relative N:P capacity (Tong *et al* 2020). Since phytoplankton growth is sensitive to stoichiometric ratios and harmful species are often favored at low N:P ratios (Frank *et al* 2020, Klausmeier *et al* 2004), the appearance of harmful algal blooms is a consequence of both total and relative nutrient loading (Anderson *et al* 2002).

Finally, although specific practices are important, understanding the cumulative (and intersecting) impacts of these practices are essential to motivate change. Quantifying the social costs of nutrients has been done for N (Sutton *et al* 2013, Sobota *et al* 2015, Keeler *et al* 2016) but not explicitly for P despite some similar damage cost work (Dodds *et al* 2008, Lürling *et al* 2016). A social cost of P analysis will be key to fully inform how and why management needs to be changed in specific contexts. Accounting for N and P together, as described here, may be critical because the benefits and costs of food security and water quality related to either nutrient interacts with the other (Lewis *et al* 2011, Hamilton *et al* 2016, Kanter and Brownlie 2019).

## 4. Conclusions

Environmental footprints offer an opportunity for consumers to reflect on how their actions are linked to nutrient use nationally and its potential effects on downstream ecosystems, and as such, they can be an important goal setting tool (Galli 2015, Leach *et al* 2016). In this work, we presented a new P footprint for the U.S. that could be matched with an existing N footprint and explored how releases of both nutrients (N:P) varied among food types and along the food production and consumption chain. We did not observe large differences in terms of ranking of food types contributing to the N and P footprints so it is likely that interventions for one nutrient will have beneficial effects on the cycling of the other. Still, there are some interventions that could create different outcomes for N than P (especially with regard to recycling technologies) so it is important that future work continue to systematically quantify N and P flows in order to be able to identify where stoichiometric ratios might cause issues. Our results show relatively large differences between N and P across components of the food system and waste management, suggesting that research on coupled nutrients and N:P ratios would help design beneficial interventions for food production and environmental quality.

## Supplementary Material

SI

## Figures and Tables

**Table 1.** Key data sources used to calculate VPFs and comparison to the established VNF approach by Leach *et al* (2012). When not mentioned, the same values (e.g. mass, proportional ratios) and calculations were used in calculating VPFs as for the VNFs. Table S1 provides further explanation of differences in data sources for specific crops.

Food item/group	Data types and assumptions required to calculate VPFs in conjunction with VNF information already available	Data sources

Food and feed crops	P content of food eaten or sold	USDA (2017)
	P content of harvested crop	IPNI (2012), state extension agency, or USDA (2017)
	P content of crop residues	Smil (1999), state extension agencies, or calculated by difference
	P content of whole plant	IPNI (2012), state extension agency, or calculated by difference
	P fertilizer application rates	State extension agencies to match N sources except when stated in table S1

Animal products	P content of products	USDA (2017)
	P content of live animal	Lorimor *et al* (2004)
	P in feed *Note that VPF values account for the full life cycle of the animal.*	National Research Council reports for each animal type (NRC 2001, NRC 2012, NRC 2000, NRC 1994)

Poultry and eggs	Nonphytate to phytate ratio in feed of 0.3/0.7	Ceylan *et al* (2003), Simons *et al* (1990) and validated with ‘Nutrient Requirements of Poultry’ by NRC (1960)

Beef and milk	Assume an average of 3 full lactation cycles, and growth in the first two years of life to add P requirements at different life periods.	Pen State Extension (2015)

## Data Availability

All data that support the findings of this study are included within the article (and any supplementary information files).

## Footnotes

8 The N footprint is centered around 2015 but the Food and Agriculture Organization Corporate Statistical Database (FAOSAT) data on the diet composition is from 2013.

9 From a nutrient budget perspective atmospheric release of P from combustion can be locally significant (Wang *et al* 2015) and atmospheric P deposition can be a relatively large input to pristine ecosystems (Stoddard *et al* 2016, Smith *et al* 2017, Amos *et al* 2018). However, the N footprint methodology does not account for atmospheric deposition and as such it would be counter-intuitive to add particulate P as a release in this paper. Compared to fertilizer inputs for food production, P inputs (and thus outputs) related to energy production (burning of biomass or production of biofuels such as ethanol (Hill *et al* 2006)) is relatively small.

10 The wastewater methodology for the N footprint was updated considerably in Cattell Noll *et al* (2020), now considering effluent TN concentrations from all reporting wastewater treatment plants in the U.S., average septic retention as well as some of the atmospheric losses associated with broad treatment types. In addition, 50% of retained biosolids are considered to be productively used and thus not a release to the environment. We use the same proportions of waste treatment technology coverage (EPA 2012), and are consistent with our approach to inputs considered as we look at more than just food.

11 The results in this section are per unit of food consumed and not reported as a proportion of the total average U.S. diet-adjusted consumer P or N footprints. As such, although poultry accounts for more of the P footprint than pork per capita, poultry actually has a lower P release per kilogram consumed.
