# Supplementary material for: The U.S. consumer phosphorus footprint: where do nitrogen and phosphorus diverge?
Source: Environ Res Lett. Author manuscript; Available in PMC 2022 Aug 19. (PMC9389546; doi:10.1088/1748-9326/aba781)

Supporting Information (SI) Materials

Table S1. Equations for calculating the A) total P footprint, B) food production P footprint, C) the wastewater P footprint. This is completely analogous to the N food footprint steps presented in Leach *et al* (submitted) except for wastewater. All data sources are listed in (Leach *et al* submitted) when we use the same as for N, and new P specific data sources are noted and listed in table 1. Each of the three main equations are then further broken down in to their components and calculation steps, notably the differences for crops and animals in the food production component of the footprint.

	Components	VPF steps	Equation representation	Specifications
A)	Total P footprint	--	Sum across food groups (food production footprint * kg food consumed) + Wastewater P footprint	The food production footprints are in kg released per kg of food, kg consumed are expressed in kg per year per capita, and release from wastewater is also kg per year per capita
B)	Food production footprint for a food item or group	--	VPF * P content of food	Note that we go from g lost per kg of food here to kg lost when calculating the Total P footprint. We do not show unit conversions in these calculations.
	Virtual phosphorus factor (VPF) for food group	--	Sum (VPF individual crops * (individual crop in US diet/total of crops considered in group in US diet))	Crops and animal products considered are listed in table S3
	**CROPS**			
	VPF individual crop	--	Sum P releases / P consumed	Unitless as it is expressed as kg P/kg P
	Sum of P releases	--	Crops: food waste, food processing, crop waste, P not taken up by crop after fertilizer application<br><br>Animals: food waste, processing waste, slaughter waste, manure release, crop waste, P not taken up by crop after fertilizer application	See figure 2 for clear numerical examples. Note that when calculated this is done for 100 units of new P, but that the ratios of P lost are based on literature values derived from the steps listed below.
	P release for crop products (by step)	1.Whole plant P uptake<br><br>2.Yield P + recycled crop residue	Avg. whole plant P /Applied P<br><br>P yield/Whole plant	Yield here refers to the harvested part of the plant. Yield weight and P content are calculated as a weighted average of state values, see Applied P for equation, but related with yield instead of fertilizer.
		3.Processing waste + recycling processing<br><br>4.Food waste	% from FAO<br><br>% from FAO	These percentages of loss/release in steps 3 and 4 are applied to the amount of P left after accounting for steps 1 and 2, see Figure 2.
	Avg. whole plant P	1 and 2	Yield P + Residue P	The ratio of residue to yield or whole plant to yield was sometimes used to determine the residue P (as it was for N).
	Applied P	1	Sum (state fertilizer recommendation * (state	See table S2 for differences in states used.

			production of crop/total of states considered for crop)	
	**ANIMALS**			
	P release for animal products (by step)	1.Feed crop whole plant P uptake	Sum across feed crops (crop value* % of crop in animal diet). See crop step 1 for each crop considered.	Feed crop considered: soybeans, corn, wheat, barley, sorghum, oats, alfalfa, and hay.
		2. Feed crop yield P + recycled feed crop residue	See crop step 1 for each crop considered.	
		3. Live animal P uptake + recycled manure	1/P_FCR	
		4. Carcass P + recycled slaughter by-products	P meat (or product) / P animal	This can also be expressed as: P content meat * meat weight / P % live animal * carcass weight
		5. Processing waste + recycled pros. Waste	% from FAO	
		6. Food waste	% from FAO	
	Feed conversion ratio (P_FCR)	3	kg weight gain or product /kg feed required * P in weight or product/P in feed required	Product here refers to milk or eggs
	Feed efficiency ratio (kg weight gain / kg feed required above)	--	Sum over lifetime (kg gained or needed * length of time in growing stage / full life expectancy)	For eggs and poultry, we multiplied P in feed required by 0.42 to account for phytate see table 1.
C)	Wastewater P footprint	--	P emitted* (1-Avg. P removal)	P 'emitted' per capita is taken from (Metson *et al* 2017) and is the sum of P emitted from excreta and detergents per person per year. Excreta and detergents are the two most important sources of P in wastewater.
	Avg. P removal in wastewater treatment	--	Sum of treatment options (removal efficiency * % population served by treatment option)	Treatment options considered were septic for those not connected to sewers, and for those connected to sewers primary, secondary, and no discharge at wastewater treatment plants.

Table S2. Differences in data sources for crop VPF compared to VNF. See Leach *et al.* (submitted) for details on the data sources.

Crops	States that did not have P application rates	States that did not have % P in edible crop	States that did not have % P in whole plant
**Tomatoes**	New York	Georgia	
**Onions**	Colorado, New York	Oregon, Colorado	Washington, Idaho
**Lettuce**		Arizona	
**Potatoes**	Arizona, Maine, New York, Virginia		
**Soybeans**	Delaware, Florida, Illinois, Missouri, Pennsylvania, South Carolina, Texas		
**Apples**			
**Grapes**			
**Oranges***			
**Watermelons**	Indiana		
**Corn**	Alabama, Arizona, California, Montana, Delaware, New Jersey, Pennsylvania, South Carolina, South Dakota, Virginia, West Virginia		
**Wheat**	Alabama, Arizona, Illinois, Kansas, Louisiana, Nebraska, Pennsylvania, South Carolina, Virginia		
**Rice**	California, Missouri, Texas		
**Barley**	Maine, Pennsylvania, Virginia, Washington		
**Sorghum**	Missouri, Texas, Arizona, Illinois		
**Oats**	Alabama, Iowa, South Carolina, Washington		
**Alfalfa**	Arizona, Arkansas, Illinois, Indiana, Kentucky, Maine, Maryland, Massachusetts, Nevada, New Hampshire, New Jersey, North Carolina, Rhode Island, Vermont, Virginia, Washington, West Virginia, Wyoming		
**Hay**	Ohio, Pennsylvania, North Carolina, Delaware, California, Arizona, Indiana, West Virginia		

*Many states recommended 0 fertilization for trees. In Florida we used a different data source for P application (Singerman 2018), but used the same assumptions about life span, tree density and yields as was done for N. *Singerman, A. (2018) University of Florida Institute of Food and Agricultural Science. Citrus Research and Education Center.* https://crec.ifas.ufl.edu/economics/

Table S3. VNF and VPF values – kg nutrient released per kg of nutrient consumed. Values in parentheses are the 95% confidence interval values. Cheese does not have a confidence interval as it uses the milk data and rice is not grown in many states and we did not have enough data to calculate such an interval. This is used in Figure S1.

Food	VNF	VPF
Animal products		
Pork	5.69 (5.94-6.55)	15.26 (15.11-15-54)
Poultry	5.08 (4.01-6.55)	15.00 (14.80-15.30)
Beef	13.44 (11.11-16.80)	54.93 (50.90-61.07)
Milk	3.90 (3.59-4.64)	2.52 (2.18-2.91)
Cheese	3.90	2.52
Eggs	4.06 (3.59-4.64)	10.98 (9.18-13.89)
Fruits - Avg	5.88 (4.39-8.53)	4.32 (2.51-54)
Apples	3.15 (2.01-5.59)	4.99 (2.78-12.57)
Oranges	6.53 (4.94-9.26)	3.04 (1.71-6.64)
Grapes	5.81 (4.03-9.44)	1.35 (0.46-4.10)
Watermelon	4.95 (4.06-6.20)	12.33 (9.55-17.01)
Vegetables - Avg	5.07 (4.33-6.07)	8.20 (6.82-10.77)
Lettuce	5.15 (4.68-5.70)	6.36 (4.05-12.47)
Tomato	5.60 (4.77-6.7)	9.65 (8.54-11.04)
Onion	3.44 (2.61-4.75)	6.49 (5.57-7.72)
Potatoes	2.62 (2.36-2.90)	2.76 (2.20-3.56)
Grains - Avg	1.70 (1.48-1.97)	1.66 (1.40-1.99)
Corn	1.13 (1.10-1.22)	1.08 (0.93-1.26)
Wheat	1.82 (1.56-2.13)	1.82 (1.51-2.21)
Rice	1.15 (1.12-1.28)	0.89 (NA)
Beans	0.49 (0.43-0.56)	0.57 (0.45-0.72)

Table S4. P released to the environment for specific food items that comprise the vegetal product food groups presented. Values in parentheses are the 95% confidence interval values. The values are used in figure 4.

Food	g P release per kg of food
Animal products	
Pork	34.03 (33.68-34.66)
Poultry	27.90 (27.53-28.45)

Beef	112.05 (103.83-124.58)
Milk	2.54 (2.20-2.94)
Cheese	12.18
Eggs	21.73 (18.17-27.51)
Fruits - Avg	0.61 (0.35-0.78)
Apples	0.55 (0.31-1.38)
Oranges	0.43 (0.24-0.93)
Grapes	0.27 (0.09-0.83)
Watermelon	1.36 (1.05-1.87)
Vegetables -Avg	2.18 (1.81-2.87)
Lettuce	1.68 (1.07-3.29)
Tomato	2.47 (2.18-2.82)
Onion	1.82 (1.56-2.16)
Potatoes	1.81 (1.44-2.33)
Grains - Avg	5.18 (4.37-6.21)
Corn	2.96 (2.53-3.44)
Wheat	6.75 (5.61-8.18)
Rice	2.59 (NA)
Beans	3.03 (2.37-3.80)

Figure S1. Relationships between N and P footprint metrics as (A) Virtual factors, (B) Release per weight of food, and (C) Release per kilocalorie of food. The trends are obviously positive. Linear regressions in all three cases have high R2 values (A. 0.71, B. 0.98, C. 0.96) with p values below 0.001, but as the data are aggregated (in the case of B. and C.) and heteroscedastic, any such statistic should be treated as just an illustration.

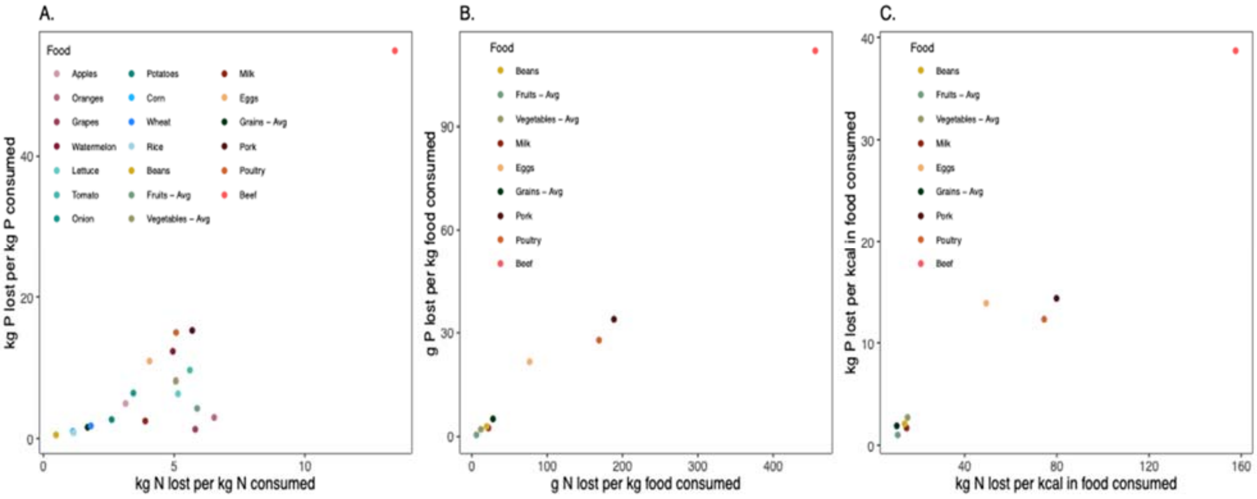

Figure S2. Comparison of grams of N and P released to the environment per gram of food consumed for (A) Animal products and major food groups and (B) Non-animal product food items that make up the categories presented in panel A. Panel A is the same data as presented in figure 4 and is presented here for scale (as per the dotted line) in relation to the values presented in panel B.

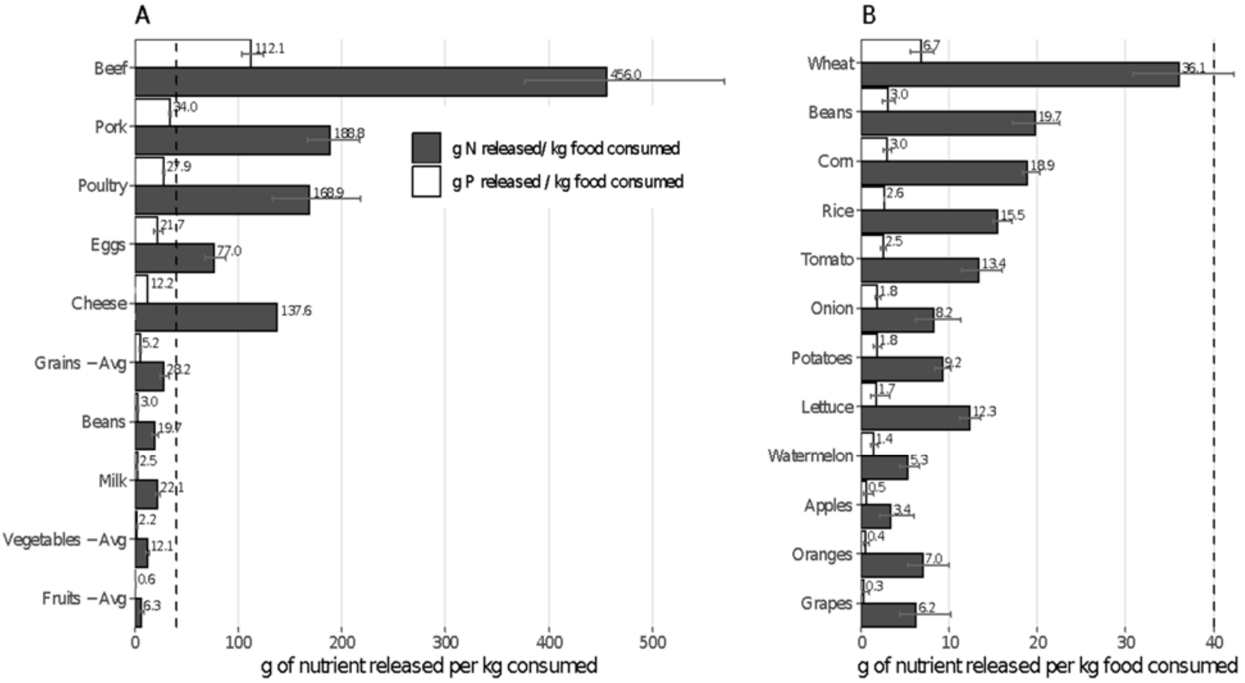

Supplement: SI [file NIHMS1648029-supplement-SI.pdf]
